# EVALUATION OF ARTHROGRYPOTIC FOOT TREATMENT: MINIMUM 10 YEARS FOLLOW-UP

**DOI:** 10.1590/1413-785220243202e275561

**Published:** 2024-06-24

**Authors:** Monica Paschoal Nogueira, Jordana Brandão Caiafa, Alessandra Porto Pereira Galdez, Rodrigo Pastick Fujino, Fernando Farcetta

**Affiliations:** 1 Hospital do Servidor Público Estadual, Department of Pediatric Orthopaedics and Limb Reconstruction, São Paulo, SP, Brazil.; 2 Hospital Maternidade Terezinha de Jesus, Juiz de Fora, MG, Brazil.; 3 Hospital São Domingos, Departament of Pediatric Orthopedics, São Luís, MA, Brazil.; 4 Fundação Altino Ventura, Recife, PE, Brazil.; 5 Associação de Assistência à Criança Deficiente, Departament of Pediatric Orthopedics, São Paulo, SP, Brazil.

**Keywords:** Arthrogryposis, Clubfoot, Quality of Life, Surgical Procedures, Operative, Artrogripose, Pé torto, Qualidade de vida, Procedimentos Cirúrgicos Operatórios

## Abstract

**Objective:**

To evaluate patients with arthrogryposis submitted to extensive surgical treatment with a minimum of 10 years of follow-up regarding the clinical and radiological aspects and the quality of life, using the 36-Item Short Form (SF-36) and the Disease-Specific Instrument (DSI).

**Methods:**

A retrospective study selected 33 patients, totaling 64 operated feet.

**Results:**

The mean age of the patients was 17.9 years (12-39 years), and the mean follow-up time was 14.8 years (11-17). Amyoplasia represented 78.7% of syndromic diagnoses. Isolated posteromedial lateral release (PMLR) was performed in 21.8% of the feet, 27.2% of which required additional bone surgery, and about 50 feet (78.1%) were submitted to PMLR, lateral column shortening, and/or talectomy. In total, 46 talectomies were performed (71.8% of the feet), out of which 44 were the first procedure of choice. SF-36 questionnaire was evaluated and showed that 93.9% of the patients did not have restrictive and disabling pain, and the same percentage considered themselves as healthy and had good expectations for the future.

**Conclusion:**

Arthrogrypotic feet are difficult to treat, require many recurrent surgical procedures, and relapses are the rule. Stiffness is a common feature of these feet, and residual deformities were frequent. ***Level of Evidence IV; Case Series, Therapeutic Studies.***

## RESUMO

**Objetivo:**

Avaliar pacientes com artrogripose submetidos a tratamento cirúrgico convencional com um mínimo de 10 anos de seguimento quanto aos aspectos clínicos, radiológicos e qualidade de vida, utilizando o questionário de 36 itens Short Form 36 (SF-36) e o Instrumento específico de Doenças (IED).

**Método:**

No estudo retrospectivo foram avaliados 33 pacientes, totalizando 64 pés operados.

**Resultados:**

A média de idade foi de 17,9 anos (12-39 anos), e o tempo médio do seguimento foi de 14,8 anos (11-17). A amioplasia representou 78,7% dos diagnósticos sindrômicos. A liberação posteromedial lateral isolada (LP MI) foi realizada em 21,8% dos pés, 27,2%, com cirurgia óssea adicional, e cerca de 50 pés (78,1%) foram submetidos a LPM (liberação póstero medial), encurtamento da coluna lateral e/ou talectomia. Foram realizadas 46 talectomias (71,8% dos pés), sendo em 44 o procedimento de primeira escolha. O questionário SF-36 evidneciou que 93,9% dos pacientes estavam sem dor restritiva e incapacitante, consideravam-se saudáveis, com boas expectativas para o futuro.

**Conclusão:**

Os pés artrogripóticos são de difícil tratamento, requerendo muitos procedimentos cirúrgicos recorrentes. A rigidez é uma característica comum desses pés e as deformidades residuais foram frequentes. Estudos futuros poderão mostrar se haverá diferença no resultado do tratamento desses pés aplicando a abordagem inicial atual, mais conservadora. ***Nível de Evidência: IV; Estudos Terapêuticos; Série de Casos.***

## INTRODUCTION

Arthrogryposis is a term used to designate signs associated with entities characterized by rigid, non-progressive contractures in two or more joints in different body areas.^1–2^ Its incidence is relatively rare, with occurrence described in the literature ranging from 1:3000 live births,^3^ with amyoplasia representing more than 1/3 of the cases, around 1:10,000 live births.^4–7^ The causes of arthrogryposis are still unknown; however, it is believed to be of multifactorial origin.^3^ The most frequent foot deformity in patients with arthrogryposis is rigid equinus, cavus, varus and adductus (78 to 90%).^2,7^ This deformity presents hypotrophy, thinner calf muscles, with fibrotic tendons and little mobility, characterized by being more severe and rigid than in the congenital clubfoot.

Treatment of arthrogrypotic feet deformities aims at obtaining plantigrade, braceable, and non-painful feet. However, the stiffness of the disease and the high risk of recurrence make the treatment of arthrogrypotic feet a challenge.^7^

One of the options of treatment for arthrogrypotic feet includes manipulation and serial casting before extensive surgical soft tissue release and talectomy. This method has satisfactory results, reducing extensive surgeries, the number of surgeries and complications.^6^ However, the stiffness found in these feet can make manipulation difficult.^3,6–8^

Conventional surgical treatment is a widely used method involving posteromedial release (PMLR), talectomy, and tarsectomy.^2,3,6,7^ PMLR is traditionally considered the first surgical method to be performed in young children and with less rigid deformities. This method consists of releasing peritalar capsules, ligaments, and tendons "à la carte" to correct deformities. Some studies suggest that PMLR alone has a higher recurrence rate.^3,6–8^

In the 1980s, Menelaus obtained good results with the talectomy in the treatment of rigid equinovarus feet in patients with arthrogryposis and those with myelomeningocele.^9^ Today, the technique is used in severe, recurrent arthrogrypotic feet with structured deformities, working as a salvage procedure, with the advantage of creating the required space to correct the deformity without tension.^7,8,10^

Other less conventional surgical methods of treatment include the Verebelyi-Ogston procedure (subchondral excision of cancellous bone from the cuboid and talus), the progressive correction of the deformity through an external fixator using the Ilizarov method, and triple arthrodesis after 10 years of age.^4,7,11–13^

Few studies in the literature assess the quality of life and long-term functional results of patients with arthrogryposis after surgical treatment of feet deformities using standardized instruments.^6,14–18^ The 36-Item Short Form (SF-36) is one of these instruments, measuring three aspects of health: functional ability, well-being, and general health.^19^ The aim of this study is to evaluate patients with arthrogryposis submitted to surgical treatment with a minimum of 10 years of follow-up regarding the clinical and radiological aspects (following the model proposed by the Clubfoot Study Group) and regarding their quality of life, using the SF-36 and the Disease-Specific Instrument (DSI).

proposed by the Clubfoot Study Group, considering clinical and radiographic parameters.

## METHODS

The retrospective study was approved by the institutional research ethics committee. Forty-two patients with arthrogryposis syndromes who underwent surgical treatment to correct feet deformities from January 1, 1974, to December 31, 2002, were included, corresponding to a minimum follow-up of 10 years. Patients were excluded from the study when there were uncertain records regarding the diagnosis and procedure performed. Thus, 33 patients (64 feet) were selected for this study.

Data was collected through an assessment questionnaire according to the model The SF 36 and DSI questionnaires were also used to evaluate the quality of life. The following aspects were evaluated:

- Demographic aspects: age, sex, and type of activity performed by the patient;
- Treatment method: the surgical treatment method to which the patient was submitted. Check on the occurrence of previous manipulation with serial casting;
- Physical Examination: the patients underwent a complete physical examination, always performed by the same examiner, including weight, height, size of the lower limb (measured from the anterior superior iliac spine to the medial malleolus), calf circumference, and foot size and width. The foot was inspected for calluses. Goniometry was performed to measure ankle dorsiflexion and plantarflexion passively, as well as the varus and valgus of the subtalar, adduction, abduction, and pronosupination of the forefoot. The strength of the tibialis anterior and posterior, triceps surae, peroneal, extensor hallucis longus, extensor digitorum, flexor hallucis longus, and flexor digitorum longus muscles was measured clinically. Patients were requested to stand in a monopodal weight-bearing position and perform repeated plantar flexions, stopping after fourteen flexions or when there was moderate pain or triceps surae fatigue.
- Radiographic examinations: weight-bearing anteroposterior and lateral radiographs of the feet were requested, and the radiographic parameters were measured by a single examiner. In the antero-posterior view, it was obtained the talocalcaneal angle, the angle between the calcaneus and the fifth metatarsal, and the angle between the talus and the first metatarsal. In the lateral view, the talocalcaneal, talus-first metatarsal, calcaneus, and first metatarsal angles were measured, as well as the angle between the first and fifth metatarsal. Degenerative changes were checked.
- Quality of life questionnaires: each patient answered a quality of life questionnaire elaborated based on the SF-36 and DSI (Disease-Specific Instrument).^(16,19)^
- The data collected was organized and analyzed using Microsoft Excel; then, the treatment methods under study were correlated with the patients’ functional status after a 10-year evolution period.

Informed consent was obtained from all patients for being included in the study, and after the ethical committee approval. Written informed consent was obtained from all patients/parents/legal guardians for publication of this manuscript and any accompanying images and videos.

## RESULTS

The study group had 11 female patients (33.4%) and 22 male patients (66.6%) with a mean age of 17.9 years (12-39 years), totaling 64 feet. The group consisted of 26 (78.78%) patients with amyoplasia (AMC), two (6.06%) patients with Larsen Syndrome, three (9.09%) patients with Moebius Syndrome, one (3.03%) patient with Streeter Syndrome, and one (3.03%) patient with Schwartz-Jampel Syndrome. (Table 1)

**Table 1:** Demographic aspects and diagnosis of patients with arthrogryposis.

Patient	Gender	Age (years)	Diagnosis
DPS	Female	18	AMC
APPS	Female	16	Larsen
MAP	Male	26	AMC
WJA	Male	11	AMC
JLF	Male	17	Schuwartz Jampel
VHR	Female	21	AMC
GAR	Male	15	AMC
MVMN	Male	13	AMC
NOC	Female	20	AMC
ALA	Male	15	AMC
NDJ	Female	18	AMC
TM	Male	15	AMC
VCS	Female	11	AMC
YSS	Female	15	AMC
EMP	Male	18	Moebius
LCM	Female	14	AMC
MAS	Male	13	AMC
KMC	Male	14	AMC
GAS	Male	16	Streeter
DPS	Male	20	Larsen
DAFS	Female	17	Moebius
VVS	Male	16	AMC
CNG	Female	17	AMC
GSC	Male	24	AMC
JCS	Female	23	AMC
BVLM	Male	25	AMC
GAM	Male	14	AMC
GTS	Male	12	AMC
MHSR	Male	15	Moebius
GRQ	Male	14	AMC
JKJS	Male	21	AMC
GIM	Male	39	AMC
MAP	Male	28	AMC

* AMC: Amyoplasia.

The mean age at the first corrective surgery of the feet was 39.31 months (3.27 years; 1-14 years). The mean follow-up time for these patients was 14.8 years (11-17 years) for each foot submitted to surgery. No patient was submitted to previous manipulation with serial casting. (Table 2)

**Table 2:** Surgical procedures in feet of patients with arthrogryposis.

Patient	Age at the first surgery (years)	Follow-up time (years)	Laterality	First surgery*	Follow-up surgery§
DPS	2	16	R	0	0
			L	3	3
APPS	1	15	R	1	1
			-	1	0
MAP	14	12	R	4	0
			L	4	0
WJA	2	11	R	3	0
			L	3	0
JLF	5	12	R	4	0
			L	1	0
VHR	3	17	R	1	1
			L	1	1
GAR	1	14	R	3	0
			L	3	0
MVMN	2	11	R	3	1
			L	3	0
NOC	8	12	R	3	0
			L	3	0
ALA	3	14	R	3	0
			L	3	0
NDJ	3	15	R	1	0
			L	2	0
TM	1	14	R	3	0
			L	3	0
VCS	2	11	R	4	0
			L	4	0
YSS	4	11	R	3	3
			L	3	0
EMP	4	18	R	3	2
			L	-	2
LCM	3	11	R	1	1
			L	3	1
MAS	1	12	R	3	1
			L	3	3
KMC	1	13	R	3	3
			L	3	0
GAS	1	13	R	3	3
			L	3	0
DPS	2	18	R	3	0
			L	2	0
DAFS	5	14	R	3	0
			L	3	0
VVS	2	14	R	3	1
			L	3	0
CNG	3	14	R	3	2
			L	3	2
GSC	6	18	R	4	0
			L	1	0
JCS	1	22	R	1	5
			L	1	5
BVLM	6	19	R	2	4
			L	2	0
GAM	3	11	R	3	3
			L	3	3
GTS	1	10	R	3	0
			L	3	0
MHSR	4	11	R	3	0
			L	3	0
GRQ	1	13	R	1	6
			L	1	6
JKJS	5	15	R	4	3
			L	4	0
GIM	4	35	R	1	3
			L	1	3
MAP	4	24	R	2	0
			L	2	0

* First surgery: 0 (No procedure); 1 (PMR); 2 (PMR+LCS); 3 (PMR + LCS+Talectomy); 4 (PMR+Talectomy).

§ Follow-up surgery: 0 (No procedure); 1 (PMR review); 2 (Debridement); 3 (Tarsectomy); 4 (Arthrodesis); 5 (LCS); 6 (Talectomy).

**Figure 1:**
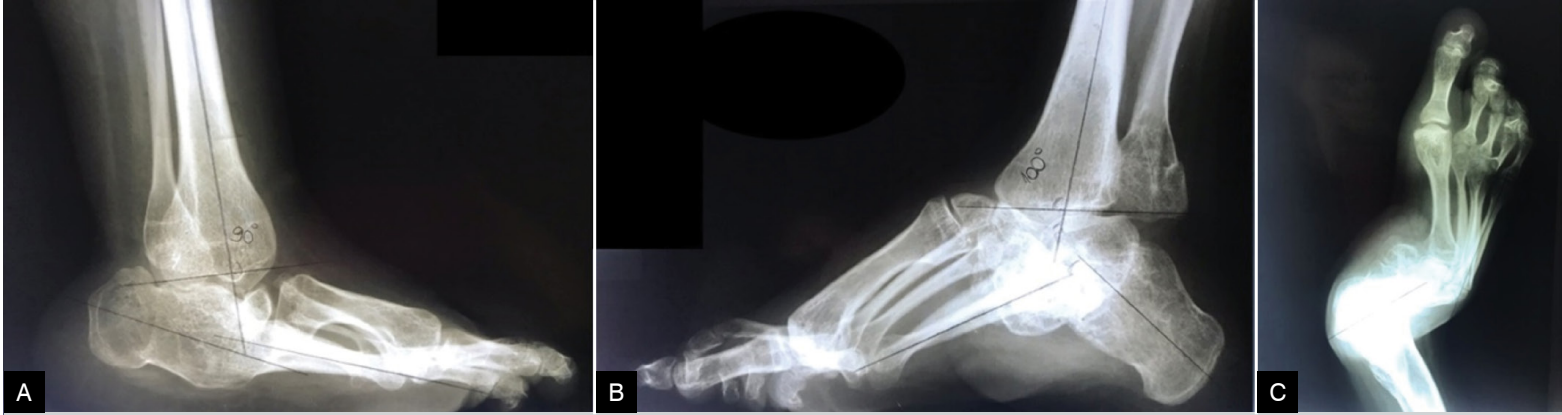
A) Lateral radiographic of the foot showing abnormal position due to talectomy in childhood, but the foot is plantigrade. B) Lateral alignment of a foot after talectomy with important cavus. C) Anteroposterior radiographic of a foot with lateral translation of the foot and adductus of the forefoot.

Isolated PMLR occurred in 14 (21.87%) feet and, later, 27.2% required additional bone surgery. Fifty feet (78.1%) underwent PMLR associated with a bone procedure, which could be the lateral column shortening and/or talectomy. Of these feet, 18% required a new bone approach, such as tarsectomy (six feet) and arthrodesis (three feet). These surgeries were performed an average of two years after the first procedure. In total, 46 (71.8% of the studied feet) talectomies were performed, out of which 44 were the first procedure of choice. PMLR associated with lateral column shortening was performed in 7.81% of the feet, PMLR associated with lateral column shortening and talectomy was performed in 56.25% of the operated feet, and PMLR associated with talectomy was performed in 14.06% of the feet. (Tables 2 and 3) Radiographic measurements were difficult due to the lack of talus in most feet. (Figure 1)

**Table 3:** Surgical aspects of feet with arthrogryposis.

Isolated PMLR (14) = 21.8%	- (4) 28.5% no more procedures were required - (6) 42.8% required additional bone surgery - (4) 28.5% required review of PMLR
PMLR + bone procedure (LCS and/or talectomy) = (50) 78.1% Of those 44 were talectomies, 68.7% of all procedures	- (9) 18% required additional bone surgery: Arthrodesis (3 feet) / Tarsectomy (6 feet) - (34) 68% no more procedures were required - (4) 8% required review of PMLR - (3) 6% required debridement

* PMLR: posteromedial lateral release. Total talectomies: (46) 71.8%, out of which (44) 68.7% first.

Based on the model proposed by the Clubfoot Study Group, the results concerning the physical aspect found in 45 (70.31%) feet were considered bad, 18 (28.12%) were terrible, and one foot (1.56%) was good. Eighteen patients (54.54%) felt pain, of these, 13 feet (72.22%) ambulate, and 5 feet (27.77%) do not ambulate. Regarding the clinical aspect of the feet, 29 (45.31%) are plantigrade, five (7.81%) have an equinus deformity, 13 (20.31%) have an adductus deformity, eight (12.5%) have a varus deformity, three (4.68%) have an equinus, cavus, varus and adductus deformity, and six (9.37%) have a valgus deformity. Sensibility was preserved in all patients assessed. There was no casting manipulation before surgical treatment. Regarding the ability to ambulate, about 10 (30.3%) patients do not ambulate, while 23 (69.69%) of them ambulate.

**Figure 2:**
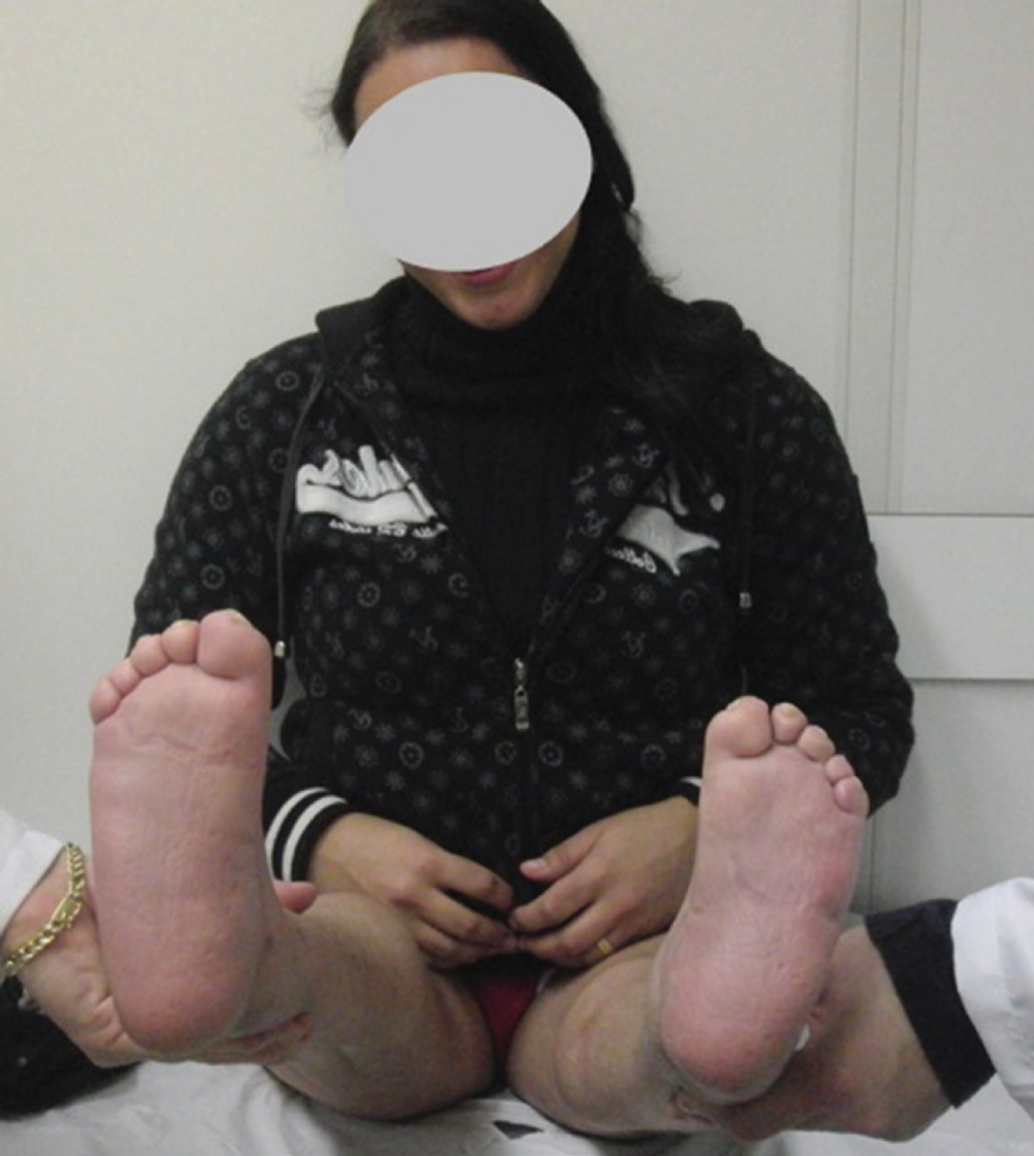
Clinical picture of a patient showing corrected, but very small size feet.

Regarding the SF-36 questionnaire, about 60.6% of the patients considered that they had some kind of limitation to perform their daily activities, 79% of patients complained that they had difficulties to buy shoes due to the very small size of their feet (Figure 2). 78.78% of the patients reported not having problems at work due to their physical disability, and 93.93% of the patients did not have disabling and restrictive pain. Only 6.06% of the patients considered that their deformities often interfere with their social activities. Furthermore, 93.93% of the patients considered themselves as healthy and with good expectations regarding future health, 84.84% considered themselves as excited and full of energy, 75.75% said they did not have any limitations due to the emotional aspects of the disability, and about 75.75% described themselves as happy. (Table 4)

**Table 4:** SF-36 scores for patients with arthrogryposis surgically treated.

Patient	Physical functioning	Physical role functioning	Pain	General health	Vitality	Social functioning	Emotional well-being	Mental Health	Total
DPS	40	50	100	97	95	75	100	72	120.4
APPS	10	100	51	75	55	87.5	66.7	40	91.1
MAP	30	25	72	87	50	100	0	56	98.6
WJA	60	75	100	72	85	100	100	92	125.4
JLF	45	50	72	92	85	50	66.7	92	117.6
VHR	40	100	41	67	40	62.5	0	32	85.5
GAR	0	100	62	95	65	100	100	76	107.2
MVMN	55	50	100	100	80	87.5	66.7	88	130.8
NOC	5	75	100	65	75	100	100	96	113
ALA	45	75	100	100	75	62.5	66.6	88	111.4
NDJ	20	75	84	52	45	100	100	44	102.8
TM	80	100	62	62	50	100	50	88	121.4
VCS	35	75	52	60	90	100	66.6	88	119.2
YSS	10	75	72	65	70	62.5	66.6	68	102.2
EMP	0	100	74	47	45	62.5	100	60	90.8
LCM	35	75	62	50	75	75	100	28	107.6
MAS	45	100	62	57	80	100	100	20	112.6
KMC	85	100	100	60	80	100	66.6	72	125
GAS	85	100	62	60	80	100	72	72	123.2
DPS	10	25	84	70	65	87.5	50	64	107.4
DAFS	70	75	62	72	40	50	66.6	60	102.6
VVS	40	75	100	67	70	75	66.6	80	109.4
CNG	50	100	64	65	40	37.5	0	36	80.4
GSC	75	100	100	60	50	100	66.6	80	122
JCS	75	0	61	65	80	62.5	0	60	97.1
BVLM	70	100	74	70	80	100	100	88	105.4
GAM	5	75	74	57	65	100	66.6	80	103.8
GTS	50	75	52	57	50	75	66.6	32	93.6
MHSR	70	100	62	75	25	50	66.6	40	98.2
GRQ	20	25	62	47	85	100	66.6	80	105.6
JKJS	20	75	100	75	65	100	66.6	80	113
GIM	35	25	82	92	80	87.5	100	64	116.4
MAP	35	25	82	92	80	87.5	100	64	115.6

## DISCUSSION

The term arthrogryposis is characterized by rigid joint contracture of two or more joints in different body areas. These clinical entities are divided into three subgroups. The first one encompasses all conditions with primary limb involvement, amyoplasia being its most common form. This disorder is characterized by rigid, symmetrical contractures such as extended elbow and feet in rigid equinus, cavus, and varus positions, which is the most frequent deformity of the feet and whose standard treatment is still surgery. The second subgroup includes those with intellectual impairment and joint contractures. The third subgroup comprises, for example, distal arthrogryposis, which may be associated with the hereditary pattern and with a normal intellectual development.^1–3,7,20,21^

Non-surgical treatment of arthrogryposis consists of physical and occupational therapy, psychological support, casting use, and stretching of the joints.^20–23^ In this study, there were no cases treated conservatively, and all cases were treated surgically.

The surgical treatment consists of correcting deformities of the lower or upper limbs with soft tissue surgery and bone procedures in childhood. Widmann et al.^3^ and Simis et al.^7^ suggest that talectomy should be the procedure of choice for the correction of equinus, cavus, and varus deformities in the feet of patients with arthrogryposis older than 1 to 2 years old and for review after soft tissue surgery. Soft tissue surgeries have a higher recurrence rate than talectomies, especially in feet with more severe deformities and in older children.^3,20,23^

There are few reports of long-term follow-up of the treatment of patients with arthrogryposis, as most prior studies only report short or mid-term results. Long-term follow-up is necessary to establish long-lasting treatment options for each affected individual, improving their quality of life.^17,20,21^

With its 36 questions, the SF-36 questionnaire measures general health results and can be used to compare the disease burden in the population and the benefits of different treatments. In the study by Dobbs et al.,^15^ the SF-36 questionnaire was used in 45 patients with congenital clubfoot treated with soft tissue surgery. Of these, eight patients underwent posterior release associated with plantar fasciotomy, while 37 were treated with posterior, subtalar, medial, and lateral releases for a mean follow-up period of 30 years, with long-term impairment of the physical function of the foot. Regarding the SF-36 questionnaire, the physical component was two standard deviations away from the average of the general population. The functional results of our arthrogrypotic patients are also low, as the surgical treated clubfeet described by Dobbs et al.^15^ Our patients were younger with follow up about 14.8 years, against 30 years in Dobbs’ paper. Dobbs patients’ poor results in the functional aspect of the foot can be underestimated, as, in some cases, there was radiographic evidence of arthrosis, but the patients were asymptomatic.

The study by Amor et al.^18^ used the Pediatric Outcomes Data Collection Instrument (PODCI) questionnaire, that were answered by the parents of 74 children diagnosed with amyoplasia with a mean age of 8.5 years. The results obtained were lower than those of children without musculoskeletal disorders in all 6 domains. During the mean follow-up period of approximately 3 years, children with amyoplasia had a statistically significant increase in the scores for upper extremity function, practicing of sports, and global function. These results showed that PODCI is useful in assessing the functional outcomes of children with amyoplasia and is sensitive to function changes over time.^18^

In our study, patients are older, and then heavier, then difficult to compare with those with amyoplasia with a mean age of 8.5 in the study by Amor et al.^18^ It is also expected than arthrogrypotic patients in our study are less functional then their non-arthrogrypotic peers. The mental component was also better, not similar to general population, but reflect that those patients possibly adapt to their restrictions. Although most of the cases had unsatisfactory results due to the clubfoot study group method (including functional and radiographic results), the results of quality of life (base on the SF-36) were satisfactory in most patients.

The multicenter study by Nouraei et al.^20^ aimed identify the long-term results of 177 adults with AMC in more than 15 countries. The study group consisted of 72% female patients with a mean age of 39 years, more than 90% of whom had involvement of the upper and lower limbs. As for the results of the SF-36 questionnaire, these patients had lower physical function and vitality scores than the general USA population.^18^ In our study, about 60.6% of the patients considered to have some kind of limitation in the performance of their daily activities, and 21.3% of the patients reported having problems at work due to their physical disability. Still, they had higher scores in others, such as the pain, vitality, social, and mental components. In a retrospective study involving six patients (12 feet), Widmann et al.^3^ evaluated the results of the primary radical soft tissue release in feet presenting equinus, cavus, and varus positions in children younger than one-year-old with arthrogryposis. Mean age at primary surgery was 7.4 months, and the mean follow-up period was 4.3 years: short-term results were encouraging.

One frequent complain was the small size of the foot, consequent of multiple extensive resections that can be a problem, not only to buy shoes, but also to maintain balance.

Despite this study, in our case, radiographic measurements were significantly impaired by the high frequency of talectomy. There was a discrepancy between clinical and radiographic findings and the patient satisfaction.

In more recent years, the less invasive Ponseti treatment has been used also for arthrogrypotic feet, with promising results.^22,24–27^ It will be interesting to compare the clinical and quality of life results with these extensive surgical methods in the long follow-up.

## CONCLUSION

Arthrogrypotic feet are difficult to treat because they usually require many surgical procedures, and relapses are the rule. The standard protocol consisted of extensive posteromedial releases, including bone resections in the first years of life. In spite of the fact that most of the cases had unsatisfactory results according to Clubfoot Study Group score (functional and radiographic results included), the results of quality of life (based on SF-36) were satisfactory in most patients.

Stiffness is a common feature of these feet, a small size foot, and residual deformities were frequent. Future studies will show whether there will be a difference in the outcome of the treatment of these feet by applying the current, more conservative initial approach.
